# Gene duplication, modularity and adaptation in the evolution of the aflatoxin gene cluster

**DOI:** 10.1186/1471-2148-7-111

**Published:** 2007-07-09

**Authors:** Ignazio Carbone, Jorge H Ramirez-Prado, Judy L Jakobek, Bruce W Horn

**Affiliations:** 1 Center for Integrated Fungal Research, Department of Plant Pathology, North Carolina State University, Raleigh, NC 27695 USA; 2 National Peanut Research Laboratory, USDA, ARS, Dawson, GA 39842, USA

## Abstract

**Background:**

The biosynthesis of aflatoxin (AF) involves over 20 enzymatic reactions in a complex polyketide pathway that converts acetate and malonate to the intermediates sterigmatocystin (ST) and *O*-methylsterigmatocystin (OMST), the respective penultimate and ultimate precursors of AF. Although these precursors are chemically and structurally very similar, their accumulation differs at the species level for Aspergilli. Notable examples are *A*. *nidulans *that synthesizes only ST, *A*. *flavus *that makes predominantly AF, and *A*. *parasiticus *that generally produces either AF or OMST. Whether these differences are important in the evolutionary/ecological processes of species adaptation and diversification is unknown. Equally unknown are the specific genomic mechanisms responsible for ordering and clustering of genes in the AF pathway of *Aspergillus*.

**Results:**

To elucidate the mechanisms that have driven formation of these clusters, we performed systematic searches of aflatoxin cluster homologs across five *Aspergillus *genomes. We found a high level of gene duplication and identified seven modules consisting of highly correlated gene pairs (*aflA/aflB, aflR/aflS, aflX/aflY*, *aflF/aflE, aflT/aflQ*, *aflC/aflW*, and *aflG/aflL*). With the exception of *A. nomius*, contrasts of mean *Ka/Ks *values across all cluster genes showed significant differences in selective pressure between section *Flavi *and non-section *Flavi *species. *A. nomius *mean *Ka/Ks *values were more similar to partial clusters in *A. fumigatus *and *A. terreus*. Overall, mean *Ka/Ks *values were significantly higher for section *Flavi *than for non-section *Flavi *species.

**Conclusion:**

Our results implicate several genomic mechanisms in the evolution of ST, OMST and AF cluster genes. Gene modules may arise from duplications of a single gene, whereby the function of the pre-duplication gene is retained in the copy (*aflF*/*aflE*) or the copies may partition the ancestral function (*aflA/aflB*). In some gene modules, the duplicated copy may simply augment/supplement a specific pathway function (*aflR/aflS *and *aflX/aflY*) or the duplicated copy may evolve a completely new function (*aflT/aflQ *and *aflC/aflW*). Gene modules that are contiguous in one species and noncontiguous in others point to possible rearrangements of cluster genes in the evolution of these species. Significantly higher mean *Ka/Ks *values in section *Flavi *compared to non-section *Flavi *species indicate increased positive selection acting in the evolution of genes in OMST and AF gene clusters.

## Background

Filamentous fungi produce a wide variety of economically important secondary metabolites (extrolites). An extrolite is any outwardly directed chemical compound that is excreted or accumulated in the cell wall of a living organism [1]. Many of these extrolite compounds are beneficial, such as antibiotics, food grade pigments, enzymes, vitamins, lipids, and various pharmaceuticals; however, others, such as mycotoxins, have deleterious effects [2]. Mycotoxins are some of the most toxic natural substances known and have been estimated to contaminate up to 25% of the world's food production [3]. Although mycotoxins are widespread, the evolutionary/ecological basis for their production is largely unknown. There are several classes of mycotoxins, based on structural and chemical properties, including polyketides (e.g. sterigmatocystin and aflatoxins; [4]), cyclic peptides, alkaloids, sesquiterpenoids (e.g. trichothecenes; [5]) and epipolythiodioxopiperazines (e.g. gliotoxin; [6]). The aflatoxin (AF) pathway is one of the most intensively studied and well characterized of the polyketide pathways. Aflatoxins are a family of toxic and carcinogenic metabolites that are responsible for contamination of agricultural crops, resulting in staggering losses to the agricultural industry and untold impact on human health worldwide [7,8].

**Figure 1:**
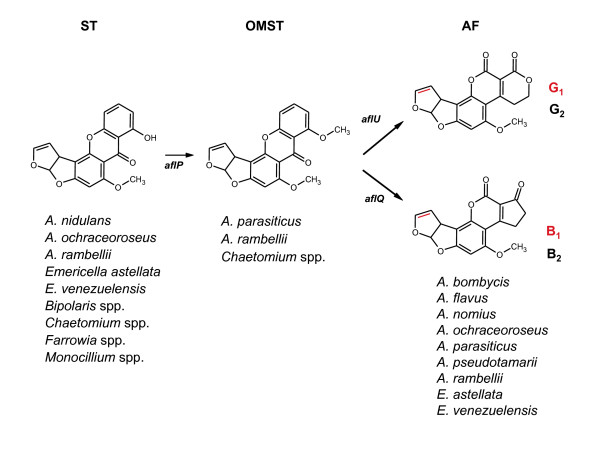
**Precursor and terminal metabolites in AF biosynthesis**. Sterigmatocystin (ST), *O-*methylsterigmatocystin (OMST) and aflatoxins (AF) are synthesized as end products by numerous ascomycetes. There are four major aflatoxins: B_1_, B_2_, G_1 _and G_2_. Aflatoxins B_2 _and G_2 _are missing the double bond (indicated in red), which is present in B_1 _and G_1_. *A. parasiticus *produces B_1_, B_2_, G_1 _and G_2_; nonaflatoxigenic *A. parasiticus *strains commonly accumulate OMST. The gene *aflU *is required for the formation of G aflatoxins [10]; *aflQ *is required for the formation of B aflatoxins [17]; and *aflP *is required for the conversion of ST to OMST [17]. *A. flavus*, *A. parasiticus*, *A. nomius *[68], *A. pseudotamarii *[69] and *A. bombycis *[68] belong to *Aspergillus *section *Flavi. Emericella *is a teleomorphic genus for the sexual stage of *Aspergillus*. *Monocillium *is an anamorphic name associated with a *Niesslia *teleomorph, also in the Phylum Ascomycota. The Ascomycota comprise highly divergent fungal lineages that span 450 million years of evolutionary history [70].

Aflatoxin-producing fungi primarily belong to *Aspergillus *section *Flavi*, which includes *A. flavus *and *A. parasiticus*, the species most responsible for aflatoxin contamination of oil-rich crops such as corn, peanuts, cottonseed, and tree nuts [9]. There are four major classes of AF, depending on the presence of the characteristic polyketide dihydro- (B_1 _and G_1_) or tetrahydro- (B_2 _and G_2_) bisfuran rings [10] (Figure 1). *A. flavus *produces aflatoxins B_1 _and B_2 _and often another mycotoxin, cyclopiazonic acid (CPA) [11,12]. Isolates differ considerably in the amount of aflatoxins produced, and populations of *A. flavus *vary in proportions of strains that produce both aflatoxins and CPA, aflatoxins alone, CPA alone, and neither mycotoxin [11]. Divergence within *A*. *flavus *has allowed for further classification of two phenotypic groups based on the morphology of the sclerotia, which are either large (L) or small (S) with a diameter of greater than or less than 400 μm, respectively [9]. Geiser *et al*. [13,14] subdivided *A. flavus *into two groups based on RFLPs of nuclear-coding genes and DNA sequences. Group I contains both L and S strains that produce aflatoxins B_1 _and B_2_, whereas Group II comprises only S strains that often produce B and G aflatoxins and represents, at least in part, an unnamed taxon. *A. parasiticus *primarily infects peanuts and is uncommon in aerial crops such as corn and cottonseed [9]. The species produces both B and G aflatoxins at generally high concentrations and nonaflatoxigenic isolates are uncommon; CPA is not produced [12]. Nonaflatoxigenic isolates of *A. parasiticus *instead often accumulate *O*-methylsterigmatocystin (OMST), an immediate precursor to aflatoxin B_1 _[12]. Section *Flavi *species other than *A. flavus *and *A. parasiticus *are mostly of minor importance to agriculture and include *A. nomius*, *A. bombycis*, and the unnamed taxon, all of which produce aflatoxins B_1_, B_2_, G_1_, and G_2_, and *A. pseudotamarii*, which produces aflatoxins B_1 _and B_2 _[15,16].

To better understand aflatoxin production in the Aspergilli, the organization, function and regulation of genes involved in AF biosynthesis has been a focus of study [17,18]. The genes in AF biosynthesis are clustered in a 70-kb DNA region and encode at least 23 coregulated transcripts under the control of the regulatory gene *aflR *[19,20]. In both the AF and sterigmatocystin (ST) gene clusters, *aflR *is a positive regulatory gene required for the transcriptional activation of most, if not all, pathway genes [21]. As shown in Figure 1, ST is produced by several fungal species, including *A. nidulans*, a model genetic system that has been used to identify the genes involved in ST biosynthesis [22]. The ST and OMST precursors are environmentally stable mycotoxins and are chemically and structurally similar to AF. The accumulation of particular extrolites of the AF biosynthetic family often differs at the species level for Aspergilli. For instance, *A*. *nidulans *synthesizes only ST, while strains of *A. ochraceoroseus *have been shown to accumulate ST and AF (Figure 1). In comparison, *Aspergillus *species in section *Flavi*, including *A*. *flavus*, *A*. *parasiticus, A*. *bombycis*, *A*. *nomius*, and *A*. *pseudotamarii*, predominantly synthesize AF. These section *Flavi *species have an identical cluster configuration, whereas gene order in *A. ochraceoroseus *is more similar to the ST cluster in *A. nidulans *[22], indicating that gene order does not determine whether ST or AF is synthesized [23]. The recent availability of the complete genome of *A. flavus *as well as other *Aspergillus *species [24-26] will allow us to further assess the role of gene duplication, recruitment and reorganization in the evolution of this important pathway.

To date eight *Aspergillus *genomes have been sequenced, including the model organism *A. nidulans *[27] and species of industrial (*A. niger *[28], *A. oryzae *[29]), medical (*A. fumigatus *[30], *A. terreus *[31]*, A. fischerianus *[32]*, A. clavatus *[33]) and agricultural (*A. flavus *[34]) importance. All genomes contain eight chromosomes but vary in their overall size and in the number of predicted genes. For example, the genomes of *A. oryzae *(37.2 Mb, 12,319 predicted genes [29]) and *A. flavus *(36.3 Mb, 13,091 predicted genes [34]) are very similar and approximately 20% larger than the genomes of *A. fumigatus *(28.8 Mb, 10,114 predicted genes [30]), *A. nidulans *(30.1 Mb, 10,701 predicted genes [27]) and *A. terreus *(29.2 Mb, 10,406 predicted genes [31]). Preliminary comparative genome analyses reveal large non-syntenous regions resulting from insertions or deletions in subtelomeric sequences, intra-molecular recombinations, variation in the number of repeated elements, tandem repeats, and gene duplicates [24]. The proximity of the AF gene cluster to the telomere in *A. flavus*, and the enrichment of secondary metabolite genes in subtelomeric regions in the Aspergilli in general, may facilitate the rapid reorganization and evolution of these genes in a species-specific fashion. This may explain the specificity of AF pathway extrolite profiles (chemotypes) for specific *Aspergillus *taxa.

The biological significance of AF chemotypes, like that of the majority of fungal secondary extrolites, is unclear. Numerous intriguing ideas regarding the function of AF pathway gene products have been offered and studies indicate that the role of these compounds in the survival of *Aspergillus *spp. may be extremely diverse [35,36]. Aflatoxins are not essential to the growth of Aspergilli under certain conditions and are not required for successful competition in AF-producing strains [35,37]. However, there may be an association between the biosynthesis of AF and developmental processes governing sporulation. Several studies have demonstrated that chemical inhibitors, mutations, and various environmental stimuli that suppress the synthesis of AF also affect or inhibit sporulation in *Aspergillus *spp. [36,38]. Although we do not fully understand the biological significance of AF extrolites, the fact that AF and ST clusters are under strong purifying selection [39] indicates that clustering is actively maintained to counteract degradation by random neutral processes. In this study, we show that gene duplication and modularity as well as positive selection are responsible for the ordering and clustering of genes in the AF pathway of *Aspergillus*.

## Results

### AF homologs and gene modules in Aspergillus

**Figure 2:**
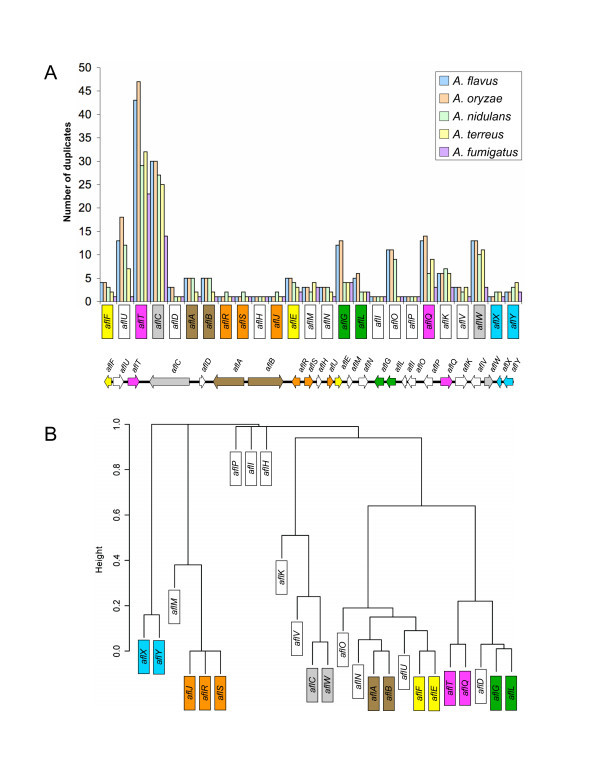
**Genome-wide tallies of aflatoxin gene duplicates, correlations among gene duplicates and inferred gene modules. A**. The histogram plot shows the total number of putative aflatoxin gene cluster duplicates on y-axis across five *Aspergillus *genomes. The gene order in the histogram follows the order of genes in the *A. flavus *cluster (see cluster schematic below histogram). **B**. Hierarchical cluster dendrogram showing the correlations among gene duplicates in Figure 2A. Correlations are based on a dissimilarity measure of (1-*r*^2^) in which correlation values are assigned "distance" values ranging from 0.0 (completely correlated, *r*^2 ^= 1) to 1.0 (completely uncorrelated, *r*^2 ^= 0). The y-axis represents the height or distance between the gene groups divided at that point. The dendrogram shows seven putative gene modules listed from left to right as: *aflX/aflY, aflJ/aflR/aflS, aflC/aflW, aflA/aflB, aflF/aflE, aflT/aflQ *and *aflG/aflL *that are highly correlated (0.80 <*r*^2 ^< 1) across the five *Aspergillus *genomes. We consider *aflR/aflS/aflJ *as correlated since only *aflH *separates *aflR/aflS *from *aflJ*. These correlated pairs are the inferred gene modules, color coded in Figure 3.

We used the predicted polypeptide sequences in *A. parasiticus *AF gene cluster as our reference sequences in TBLASTN and TBLASTX comparisons of the *A. nidulans, A. fumigatus*, *A. flavus, A. terreus*, and *A. oryzae *genome databases. The genomes for *A. nidulans, A. fumigatus, A. flavus, A. terreus *and *A. oryzae *provide 13X, 11X, 10X, 11X and 9X sequence coverage, respectively [24-26,34]. Table 1 summarizes the map location (chromosome or contig), E-value, percent coverage, and gene orientation, which is the direction of transcription depending on whether the top (+) or bottom (-) strand is being transcribed, for the two best homologs across all five *Aspergillus *genomes. The total number of putative duplicates for each cluster gene is plotted in Figure 2A.

**Table 1:** Summary of aflatoxin gene duplications, cut-off values and orientations of homologs across five *Aspergillus *genomes.*

		*A. flavus*					*A. oryzae*					*A. nidulans*					*A. terreus*					*A. fumigatus*				
Gene	Gene size (bp)	Chromosome	E-value	% Coverage	Strand	Duplicates	Chromosome	E-value	% Coverage	Strand	Duplicates	Chromosome	E-value	% Coverage	Strand	Duplicates	Contig	E-value	% Coverage	Strand	Duplicates	Chromosome	E-value	% Coverage	Strand	Duplicates

*aflF*	1149	5	0	97	-	4	5	0	99	-	4	7	1.0E-134	99	-	3	2	5.0E-99	89	-	2	7	6.0E-19	29	+	1
		2	1.0E-123	97	-		2	1.0E-120	91	-		3	1.0E-88	87	+		14	3.0E-99	87	+						
*aflU*	1497	3	0	96	+	13	3	0	77	+	18	8	5.0E-48	97	+	12	4	1.0E-114	88	-	7	5	6.0E-48	60	+	1
		6	1.0E-115	98	-		6	1.0E-98	87	-		5	1.0E-46	96	-		5	1.0E-104	99	-						
*aflT*	1545	3	0	100	+	43	3	0	88	+	47	8	1.0E-136	99	+	29	10	1.0E-162	93	+	32	6	1.0E-157	98	-	23
		4	1.0E-162	93	+		4	1.0E-162	93	+		8	1.0E-153	95	-		4	1.0E-141	80	+		1	1.0E-143	95	-	
*aflC*	6330	3	0	100	-	30	3	0	100	-	30	4	0	100	-	27	8	0	83	+	25	2	0	96	-	14
		1	0	100	+		1	0	100	+		2	0	98	+		1	0	97	+		4	0	70	+	
*aflD*	816	3	1.0E-162	100	+	3	3	1.0E-167	100	+	3	4	6.0E-65	95	+	1	10	9.0E-37	79	-	1	5	4.0E-28	81	+	1
		6	6.0E-35	84	-		6	9.0E-36	84	-																
*aflA*	5016	3	0	98	-	5	3	0	100	-	5	8	0	96	+	5	6	0	94	+	2	3	0	91	-	1
		8	0	91	-		8	0	90	-		4	1.0E-133	95	-		13	0	90	-						
*aflB*	5667	3	0	98	+	5	3	0	100	+	5	4	0	97	+	5	6	0	78	-	2	3	0	42	+	1
		8	0	100	+		8	1.0E-145	98	+		8	0	97	-		13	1.0E-176	98	+						
*aflR*	1335	3	0	100	-	1	3	0	100	-	1	4	2.0E-30	96	-	2	12	7.0E-15	85	+	1	4	3.0E-15	68	+	1
												3	6.0E-27	97	+											
*aflS*	1317	3	0	100	+	1	3	0	100	+	1	8	6.0E-61	73	+	2	12	2.0E-39	90	+	1	4	9.0E-54	51	+	1
*aflH*	837	3	1.0E-174	100	+	1	3	1.0E-172	100	+	1	4	4.0E-99	79	+	1	9	2.0E-06	31	-	1	2	9.0E-16	57	-	1
*aflJ*	945	3	0	100	+	1	3	0	100	+	1	5	3.0E-76	95	-	2	1	5.0E-96	96	-	1	3	3.0E-12	33	-	1
												4	1.0E-75	90	+											
*aflE*	1167	3	0	100	+	5	3	0	100	+	5	4	1.0E-149	95	-	4	14	1.0E-113	87	+	3	2	1.0E-113	87	-	2
		5	1.0E-119	84	-		5	1.0E-119	85	-		8	1.0E-101	88	-		2	1.0E-91	87	-		2	1.0E-113	87	-	
*aflM*	789	3	1.0E-144	100	+	3	3	1.0E-153	100	+	3	4	1.0E-115	98	-	2	5	9.0E-36	96	+	4	2	6.0E-36	93	-	3
		8	2.0E-24	98	-		8	1.0E-25	98	-		8	1.0E-95	97	+		8	4.0E-32	96	-		7	1.0E-27	77	+	
*aflN*	1479	3	0	90	+	3	3	0	100	+	3	4	1.0E-101	95	-	3	13	7.0E-53	80	+	2	3	1.0E-15	71	+	1
		8	1.0E-90	92	+		8	1.0E-90	92	+		5	1.0E-63	99	+		11	1.0E-39	91	-						
*aflG*	1488	3	0	92	-	12	3	0	100	-	13	4	0	95	-	4	3	2.0E-47	94	+	4	4	2.0E-50	88	-	4
		1	3.0E-50	83	+		1	1.0E-49	89	+		1	4.0E-30	87	-		13	5.0E-47	94	-		7	5.0E-42	88	-	
*aflL*	1503	3	0	100	-	5	3	0	100	-	6	4	0	96	-	2	3	8.0E-56	93	+	2	7	1.0E-63	90	-	2
	1503	3	3.0E-66	89	+		3	3.0E-66	89	+		1	6.0E-34	95	-		10	5.0E-55	85	+		4	5.0E-57	89	+	
*aflI*	858	3	1.0E-164	99	-	1	3	1.0E-159	100	-	1	4	9.0E-84	85	-	1	12	2.0E-13	36	-	1	4	1.10E-02	14	-	1
*aflO*	1161	3	0	100	-	11	3	0	100	-	11	4	0	98	-	9	1	2.0E-33	43	+	1	7	8.0E-24	73	-	1
		5	2.0E-66	84	-		5	2.0E-66	87	-		4	5.0E-50	81	+											
*aflP*	1257	3	0	100	-	1	3	0	100	-	1	4	1.0E-17	67	-	1	1	3.0E-20	66	-	1	5	1.0E-87	81	-	1
*aflQ*	1587	3	0	100	+	13	3	0	100	+	14	7	1.0E-111	91	-	6	11	1.0E-132	91	+	9	8	1.0E-28	88	+	3
		4	1.0E-117	89	+		4	1.0E-115	91	+		6	4.0E-66	90	+		3	1.0E-110	90	+		8	1.0E-28	78	+	
*aflK*	1932	3	0	100	+	6	3	0	100	+	6	4	0	93	+	7	2	2.0E-63	87	-	6	3	2.0E-68	89	+	3
		4	1.0E-55	86	-		4	6.0E-56	86	-		6	1.0E-100	89	+		11	6.0E-59	70	-		2	2.0E-50	89	+	
*aflV*	1527	3	0	100	-	3	3	0	100	-	3	4	0	93	+	2	4	1.0E-24	75	-	3	4	6.0E-13	84	-	1
		1	3.0E-59	89	+		1	3.0E-59	90	+		1	8.0E-54	90	-		13	2.0E-23	83	+						
*aflW*	1446	3	0	99	+	13	3	0	98	+	13	4	0	98	+	10	10	6.0E-50	96	+	11	6	4.0E-48	96	+	3
		5	2.0E-55	96	-		5	2.0E-55	95	-		3	9.0E-50	94	-		2	3.0E-41	97	+		6	6.0E-34	92	+	
*aflX*	801	3	1.0E-172	100	-	1	3	1.0E-173	100	-	1	4	3.0E-82	77	-	2	7	6.0E-46	95	+	2	4	1.0E-67	94	-	1
												8	3.0E-32	94	-		12	3.0E-33	94	-						
*aflY*	1488	3	0	99	-	2	3	0	100	-	2	4	1.0E-140	96	+	3	12	4.0E-30	85	+	4	2	1.0E-26	73	-	2
		4	2.0E-31	85	+		4	2.0E-31	85	+		8	1.0E-50	83	-		2	2.0E-24	79	+		4	1.0E-26	70	-	

*For *aflI *in *A. fumigatus *and *aflH *in *A. terreus*, the E-values are greater than the cut-off value for homology searches (E < 10^-8^).

**Figure 3:**
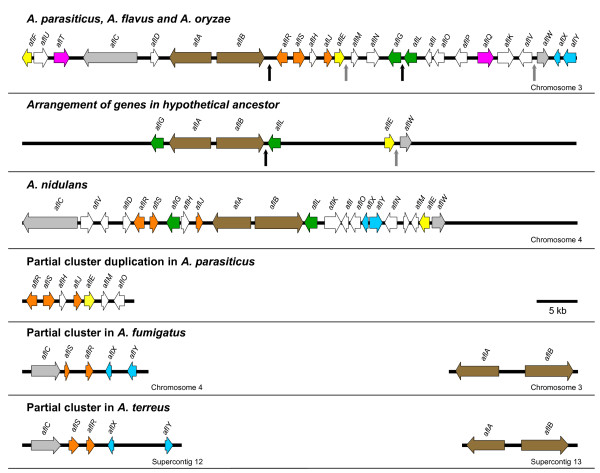
**Gene module reorganization in complete clusters and modularity in partial clusters**. The cluster schematic shows the chromosomal location, gene order and direction of transcription of genes in ST, AF and partial gene clusters. To simplify comparisons among AF and ST clusters we adopt the new AF gene nomenclature throughout [17]. The seven inferred gene modules are color coded. The arrows in the cluster at the top indicate the location of noncontiguous recombination blocks in the *A. parasiticus *gene cluster [40]. The intergenic regions indicated by the black arrows share a common evolutionary history and can be concatenated into a single contiguous block such that *aflB *and *aflL *are adjacent in a hypothetical ancestor. Similarly the intergenic regions shown with grey arrows can be reunited such that *aflE *and *aflW *are adjacent. Overall this reorganization mirrors the order of these genes in the *A. nidulans *ST cluster and highlights the importance of gene module reorganization in the evolution of AF and ST clusters. A partial cluster duplication has been reported for some strains of *A. parasiticus *[71]. Syntenic partial clusters of five genes (*aflC, aflS, aflR, aflX *and *aflY*) were identified in *A. fumigatus *and *A. terreus*.

In general, there is conservation of gene order and direction of transcription for specific groups of two or more AF pathway genes. We tested the hypothesis that genes showing a similar pattern of copy number across species have been duplicated together in groups that we term 'gene modules'. If the average copy number was less than two across all five genomes then we also considered the proximity of genes in inferring gene modules. Correlated genes that are not genomically proximate reflect historical modules that have undergone recent reorganization. The dendrogram in Figure 2B shows that gene copy number for groups of two or three AF cluster genes is significantly correlated (*P *< 0.05; 0.8 <*r*^2^*<*1). These highly correlated genes or modules, which may function as distinct biological units in AF biosynthesis, are color coded in Figure 3.

We identified seven putative gene modules across the five *Aspergillus *genomes. Not all genes in modules are syntenic across all genomes. There is conservation in gene order and direction of transcription for 1) all genes in the *A. parasiticus*, *A. flavus *and *A. oryzae *AF gene clusters, 2) modules with two genes (e.g., *aflR/aflS, aflA/aflB*) in the *A. nidulans *ST cluster and the *A. parasiticus, A. flavus, A. oryzae *AF clusters, and 3) at least two cluster genes (*aflA/aflB*) in *A. fumigatus *and *A. terreus *genomes (Figure 3). Syntenic partial clusters of five genes (*aflC, aflS, aflR, aflX *and *aflY*) were identified in *A. fumigatus *and *A. terreus*. Both the *A. fumigatus *partial cluster and the *A. nidulans *ST cluster reside on chromosome 4 while the *A. parasiticus*, *A. flavus *and *A. oryzae *AF gene clusters are located near the telomere of chromosome 3. From these data alone, the phylogenetic relationships among *A. fumigatus, A. terreus, A. nidulans *and section *Flavi *species can not be fully resolved, but the observed synteny in the partial clusters of *A. fumigatus *and *A. terreus *may indicate that similar evolutionary mechanisms have influenced the evolution of these clusters. Gene modules that are contiguous only in the AF clusters of certain species may arise from gene reorganization that reunites previously separated genes. A striking example is *aflG/aflL*, which is contiguous only in the cluster of section *Flavi *species, suggesting either recruitment from other genomic locations or reorganization of cluster genes from an ST ancestor (Figure 3). Population genetic analyses of molecular sequence variation in the aflatoxin gene cluster of *A. parasiticus *support the latter hypothesis [40]. Other putative gene modules *aflF/aflE, aflT/aflQ*, and *aflC/aflW *are separated by more than 35 kb in ST and AF gene clusters.

There was no evidence of partial clustering of two or more gene modules residing outside the AF and ST clusters. Thus, we focused on the gene module itself and examined the orientation and separation of genes in modules residing outside the cluster (Table 1). Our definition of a gene module is independent of the physical proximity of genes. Even gene modules that are syntenic in all species clusters vary in their degree of synteny when residing outside of the cluster. For example, in *A. flavus*, the two *aflA/aflB *gene modules that map to chromosome 3 but reside outside the cluster are nonsyntenous. In one module, the *aflA *and *aflB *genes are separated by 30 kb and in the other module by approximately 40 kb. Other gene modules residing outside the cluster show a high degree of synteny. For example, a copy of *aflF/aflE *on chromosome 7 of *A. nidulans *(not shown in Table 1) is contiguous and *aflF *and *aflE *are separated by less than 1 kb, comparable to the distance separating contiguous gene modules in the cluster. In some cases the orientation of genes in modules residing outside the cluster in one species matches the configuration of genes in a different species. For example, a copy of the *aflX/aflY *module on chromosome 8 of *A. nidulans *(Table 1) has the same order and gene orientation as *aflX/aflY *found in the AF clusters of section *Flavi *species (both genes negatively transcribed). This conservation further supports the vertical transmission of these modules.

### Species-specific adaptation

Initially we observed conserved syntenic relationships among AF gene clusters that mirrored phylogenetic species groupings. For example, within section *Flavi*, all species show high conservation in gene order and direction of transcription. A second grouping that includes *A. fumigatus *and *A. terreus *has conserved partial clusters. The apparent outlier, *A. nidulans*, shares gene modules with both groups as well as local rearrangements of modules, giving rise to a unique cluster configuration that is intermediate in size to partial and full gene clusters. Indeed, if cluster configuration is indicative of higher-order phylogenetic relationships among these species, then molecular variation in cluster genes would be expected to track with the underlying phylogeny and could potentially also be linked to evolutionary/ecological processes of species adaptation and diversification.

**Figure 4:**
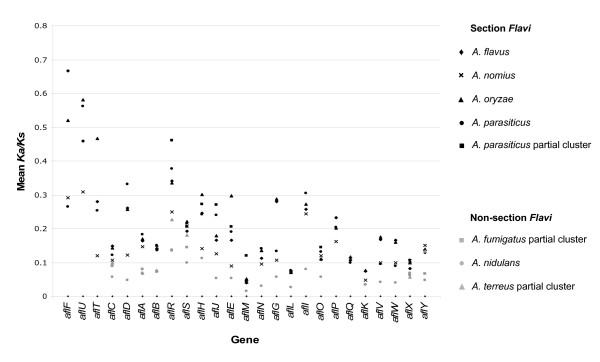
***Ka/Ks *analysis for AF cluster orthologs**. Plot of mean *Ka/Ks *values on y-axis for cluster orthologs in *A. parasiticus, A. flavus, A. oryzae*, *A. nomius *and *A. nidulans*, as well as for putative partial clusters in *A. parasiticus, A. fumigatus *and *A. terreus*. Mean *Ka/Ks *values for each gene are based on all pairwise comparisons with each species designated separately as the reference sequence.

The impact of positive (adaptive) or negative (purifying) selection on putative orthologs in full or partial AF clusters in *Aspergillus *was determined by calculating the ratio of amino acid (*Ka*) to synonymous (*Ks*) substitutions using GenomeHistory [41]. The magnitude of the *Ka/Ks *ratio provides evidence of genes under strong functional constraints (*Ka/Ks *< 1) or undergoing adaptive evolution (*Ka/Ks *> 1). We considered a linear model that parameterizes the selective pressure *(Ka/Ks*) on gene clusters in terms of variation across all cluster genes and species. Contrasts between section *Flavi *and non-section *Flavi *species showed significant differences in mean *Ka/Ks *values (*t *= -6.78, *P *< 0.0001), and mean *Ka/Ks *values were significantly higher for section *Flavi *species than for non-section *Flavi *species (Figure 4). With the exception of *A. nomius*, pairwise contrasts among section *Flavi *species indicated no significant differences in mean *Ka/Ks *values for *A. parasiticus*, the *A. parasiticus *partial cluster duplication, *A. flavus *and *A. oryzae*. Similarly, there were no significant differences in mean *Ka/Ks *values among non-section *Flavi *species; however, mean *Ka/Ks *values for *A. nomius *were more similar to *Ka/Ks *values of partial clusters in *A. fumigatus *and *A. terreus *than to the *A. nidulans *cluster (*t *= 3.13, *P *< 0.01).

## Discussion

Our systematic genomic searches for duplicated AF cluster homologs followed by correlation analysis revealed seven putative gene modules: *aflA/aflB, aflR/aflS, aflX/aflY, aflF/aflE, aflT/aflQ*, *aflC/aflW*, and *aflG/aflL*. Not all the genes in these modules are contiguous across all five *Aspergillus *species. The strong correlation observed among noncontiguous members of gene modules that are sometimes separated by more than 30 kb is consistent with vertical transmission but argues against horizontal transfer, which would require a simultaneous transfer of unlinked copies to all species, a highly unlikely event. Further evidence in support of vertical transmission is the report of putative homologs of AF genes in the pine needle pathogen, *Dothistroma septosporum *(previously known as *D. pini; *[42,43]) and in the plant pathogen, *Cercospora nicotianae *[44]. Among the putative AF orthologs identified in *D. septosporum*, the gene with the highest percent amino acid identity, *dotA*, shows 80% similarity to *aflM *of *A. parasiticus *[42,43]. In *C. nicotianae*, the CRG1 N-terminus zinc finger motif is homologous to the zinc finger domains of various regulatory proteins, including *aflR *of *Aspergillus *species [44]. The existence of *aflM *and *aflR *homologs in two ascomycete classes (Dothideomycetes and Eurotiomycetes) further argues against horizontal gene transfer and suggests that high sequence identity is the result of descent from a common ancestor and strong purifying selection.

It has been long proposed that metabolic gene clusters may be transferred horizontally between organisms [45,46]; however, direct experimental evidence that horizontal gene transfer maintains clustering in fungi is lacking. The phylogenetic evidence in support of horizontal gene transfer is also weak. In fact, phylogenetic analysis of polyketide synthases among fungal species indicates that gene duplications and losses can explain the data equally well and there is no need to invoke horizontal gene transfer [47]. Our comparative analyses suggest that intra-genomic reorganization followed by vertical descent and gene loss is a more plausible mechanism and may explain the variation in chemotype profiles for different *Aspergillus *species. For example, *A. nomius *and *A. bombycis *produce both B and G aflatoxins whereas *A. flavus *synthesizes predominantly B aflatoxins. Species producing only B aflatoxins may have evolved due to the loss of genes required for the synthesis of G aflatoxins [10]. Specifically, *aflU*, which is missing or nonfunctional in *A. flavus *isolates, may be important in G aflatoxin production since the disruption of *aflU *in *A. parasiticus *results in the production of only B aflatoxins [10]. Indeed, the location of the AF cluster in the telomeric region of *A. nidulans, A. flavus *and *A. oryzae *would facilitate gene loss as well as recombination, DNA inversions, partial deletions, translocations and other genomic rearrangements [39,48-50].

Comparative analysis of complete and partial AF clusters across five *Aspergillus *species revealed a striking modular organization of pathway genes. We hypothesize that gene modules that are contiguous in one species and noncontiguous in others are the result of rearrangements in an ancestral species. For example, four cluster genes separate *aflG *and *aflL *in *A. nidulans *whereas *aflG *and *aflL *are contiguous in section *Flavi *gene clusters. If *aflG *and *aflL *underwent reorganization in the evolution of section *Flavi *species from an ancestor with a cluster configuration similar to *A. nidulans*, this suggests that the arrangement of *aflG *and *aflL *in the cluster does not determine whether ST or AF is synthesized. Indeed, *A. ochraceoroseus *has a cluster configuration very similar to *A. nidulans *and can synthesize both ST and AF [23]. Furthermore, gene modules need not be contiguous or clustered to remain functional. For example, an *aflR *duplicate that resides outside the cluster in some *A. parasiticus *strains has been reported to regulate AF biosynthesis [51], and *aflR *in the cluster can control the expression of other genes within the genome [52]. In contrast, *aflD *is not expressed at native levels when moved outside of the *A. parasiticus *cluster, indicating that clustering does play an important role in regulating the expression of some AF biosynthetic genes [53].

Several hypotheses have been proposed to explain clustering in fungal genomes. Clustering can be a means of optimizing coregulation of genes, although clustering is not a prerequisite for coregulation as evidenced by the discovery of global regulatory genes of secondary metabolite clusters in *Aspergillus *spp. [54,55]; conversely, regulatory genes contained within gene clusters can control the expression of other genes outside of the clusters [52]. Selection acting on the cluster itself has also been invoked to explain the presence of gene clusters. In this case, the selection is independent of the selective advantage that the products of the pathway confer on the host organism [45]. This "selfish cluster" hypothesis postulates that horizontal gene transfer is an important mechanism for propagating and maintaining gene clusters in eukaryotes, reminiscent of the "selfish operon" hypothesis proposed in prokaryotes [56]. Other hypotheses postulate coadaptation and possibly gene duplication and differentiation as driving forces in gene cluster evolution [56].

Several mechanisms may have been important in the evolution and retention of AF gene modules. Gene modules may have arisen from duplications of a single gene whereby the copy retained the function of the pre-duplication gene, as observed with the *nor *reductase genes, *aflF*/*aflE *[17]. Alternatively, gene modules may have undergone subfunctionalization in which copies partition the ancestral function, as with the fatty acid synthases, *aflA/aflB *[57,58]. Other gene modules comprise genes that augment a specific pathway function, as exemplified by *aflR/aflS*, the pathway-specific transcription activator and enhancer [59], and *aflX/aflY*, the genes required for the conversion of versicolorin A to demethylsterigmatocystin [60]. The functional relationships among genes in noncontiguous modules *aflT/aflQ *and *aflC/aflW *are unknown but could include neofunctionalization, an adaptive process in which a completely new function has evolved for the duplicated copy. In addition to these localized gene duplication events, we cannot rule out a whole-genome duplication in an *Aspergillus *ancestor; conclusive evidence for this will require further analysis of gene duplicates among several genomes [61].

Adaptive processes may extend beyond gene modules to entire clusters of genes. We hypothesize that gene cluster evolution was driven by selection for new chemotypes, in this case, OMST and AF from an ST ancestor. If AF gene clusters evolved by the reorganization and recruitment of additional genes in an ST ancestor, then partial clusters synthesizing intermediate compounds might represent the earliest or ancestral clusters. Are the partial clusters identified in *A. fumigatus *and *A. terreus *functional and are they the building blocks for larger clusters? Phylogenetic studies with sufficient taxon sampling suggest that *A. fumigatus *and *A. terreus *are ancestral to section *Flavi *[24,62]. Both *A. fumigatus *and *A. terreus *have the *aflA/aflB *gene modules and partial clusters of five genes: *aflC, aflS, aflR, aflX *and *aflY*. It has been speculated that a partial cluster consisting of *aflC, aflR, aflS, aflA*, and *aflB *would have allowed an *Aspergillus *ancestor to stabilize the polyketide to an anthraquinone [16]. Anthraquinones are colorful polycyclic aromatic hydrocarbons that accumulate in spores and may aid in their dispersal via arthropods and protection from predation [16]. Spore dispersal would impart increasing selective pressures on fungi to synthesize an arsenal of polyketide derivatives to facilitate the colonization of diverse and sometimes hostile environments. Indeed, our estimates of mean *Ka/Ks *values were significantly higher in section *Flavi *than in non-section *Flavi *species, indicating increased positive selection acting on genes in OMST and AF clusters relative to the ST cluster in *A. nidulans *and partial clusters in *A. fumigatus *and *A. terreus*.

Overall *Ka/Ks *ratios for AF homologs were less than one for both section *Flavi *and non-section *Flavi *species, indicating an ongoing process of purifying selection acting to eliminate mutations that have deleterious effects on chemotype biosynthesis. Our estimates of *Ka/Ks *were consistent with values reported by Ehrlich and coworkers in AF and ST clusters [39]. Within section *Flavi*, our micro-evolutionary analyses in *A. parasiticus *[40] suggest that the most recent common ancestor (MRCA) either produced high levels of G_1 _relative to B_1 _or was an OMST producer. Since no species is known to produce only G aflatoxins, a more likely hypothesis is that the MRCA of section *Flavi *was a B and G aflatoxin producer and that selection has been acting on the G_1_/B_1 _ratio. One possible MRCA is *A. nomius*, a clear outgroup to section *Flavi *species that produces both B and G aflatoxins [63,64]. Another possibility is the unnamed taxon, which can also synthesize B and G aflatoxins [39]. The differences in aflatoxins produced by different species most likely represent a complex process that involves purifying and positive selection acting on a B and G producing ancestor; specific demographic, environmental and/or evolutionary processes in populations that maintain or break down AF gene clusters; and the actions of specific genes that are involved in AF pathway regulation [52] or other global regulatory genes of secondary metabolite clusters [54,55]. If the AF cluster arose from rearrangements of gene and/or gene modules in an ancestral *Aspergillus *species, then the signature of cluster reorganization may still be evident in descendent species. Preliminary analysis of molecular variation in the aflatoxin gene cluster of *A. parasiticus *[40] provides evidence for cluster reorganization from an ST ancestor, as well as evidence for recombination, balancing selection and chemotype-specific adaptation.

## Conclusion

Based on correlation and cluster analyses of AF gene cluster duplicates across five *Aspergillus *species, we inferred seven gene modules: *aflA/aflB, aflR/aflS, aflX/aflY, aflF/aflE, aflT/aflQ*, *aflC/aflW*, and *aflG/aflL*. Our definition of a module includes the possibility that genes may become separated after their duplication and we hypothesize that differences in gene order between AF and ST clusters may be the result of gene reorganization in an ST ancestor. Gene duplication and vertical transmission appear to be the driving forces in the evolution and retention of AF gene modules across all five *Aspergillus *species. Gene modules may arise from duplications of a single gene, whereby the copy retains the function of the pre-duplication gene (*aflF*/*aflE*) or partitions the ancestral function (*aflA/aflB*). Alternatively, the duplicated copy may simply augment or supplement a specific pathway function (*aflR/aflS *and *aflX/aflY*) or evolve a completely new function as exemplified with *aflT/aflQ *and *aflC/aflW*. Significantly higher mean *Ka/Ks *values in section *Flavi *compared to non-section *Flavi *species is evidence of adaptation and increased positive selection acting on genes in OMST and AF clusters relative to the ST cluster in *A. nidulans *and partial clusters in *A. fumigatus *and *A. terreus*. Whether patterns of gene duplication and modularity in the aflatoxin gene cluster are further influenced by evolutionary processes in populations that maintain or break down AF gene clusters are unknown and an important area of further research.

## Methods

### AF homologs in Aspergillus

Genes were considered orthologous if they satisfied the following criteria: 1) at least two genes were syntenic, 2) the genes were the best reciprocal TBLASTN and TBLASTX hits with an E-value less than 10^-8^, and 3) the genes showed amino acid similarities of approximately 40% or greater and at least 70% of the amino acids could be aligned to the reference sequence. Results from BLAST searches were further parsed to determine if cluster genes were single copy or duplicated. The total number of putative gene copies within each genome was determined using the above criteria with two exceptions: 1) reciprocal BLAST hits were not performed, and 2) an E-value less than 10^-20 ^was used when there was more than one copy to decrease the number of false positives.

### Gene modules

We identified as modules any group of two AF cluster genes that are highly correlated (*P *< 0.05; 0.8 <*r*^2^*<*1) across the five *Aspergillus *genomes. We assessed correlation and clustering using Kendall's coefficient of concordance implemented in the R statistical package [65]. This was followed by a series of *F-*tests to test the null hypothesis of no relationship between each pair of highly correlated genes [66]. Significance thresholds were Bonferroni-corrected by dividing by the total number of tests performed.

### Species-specific adaptation

Phylogenetic studies support a basal placement of *A. nidulans *and *A. terreus *relative to *A. fumigatus *and section *Flavi *species [24,62]. Because all species in section *Flavi *share a recent common ancestor and are related to non-section *Flavi *species by an underlying phylogeny, we cannot assume independence among species with respect to their *Ka/Ks *values. We therefore tested whether there was a difference in mean *Ka/Ks *values between AF cluster homologs in section *Flavi *versus non-section *Flavi *species by constructing a linear model to account for variation between genes. This model can be written as *Ka/Ks *= mean of all *Ka/Ks *values + gene effect + species effect + error.

We tested the null hypothesis that there is no difference in mean *Ka/Ks *between species in section *Flavi *and non-section *Flavi *by computing and testing arbitrary species contrasts. For example, a contrast of the form c(-3,5,5,-3,-3,-3,-3,5) where the species order is *A. flavus, A. fumigatus*, *A. nidulans, A. nomius, A. oryzae, A. parasiticus *partial cluster, *A. parasiticus*, and *A. terreus *would compare the mean *Ka/Ks *of the section *Flavi *species with the mean *Ka/Ks *of the non-section *Flavi *species. In the above contrast, all species in section *Flavi *are assigned the same numerical value (-3) and non-section *Flavi *species are given a different number (5) such that the sum of both groups in the contrast is zero (-3 × 5 + 5 × 3). Contrasts were computed using the fit.contrast function implemented by Gregory R. Warnes in the gmodels package in R [67]. The function returns a matrix containing the estimated regression coefficients, standard errors, *t*-values and two-sided *P*-values. A significant test result may indicate a difference in selective constraints on amino acid substitutions or adaptive evolution between the two species groups.

## Authors' contributions

IC and JHRP conceived the study and contributed equally to the acquisition, statistical analysis and interpretation of data. JLJ and BWH were involved in drafting the manuscript and revising it critically for important intellectual content. All authors read and approved the final manuscript.
